# MeCoLog: Meta-contrastive learning for cross-system few-shot log-based anomaly detection

**DOI:** 10.1371/journal.pone.0356907

**Published:** 2026-08-27

**Authors:** Manh Tuan Nguyen, Tuan Phong Tran, Le Dinh Trang Dang, Van Loi Cao

**Affiliations:** Institute of Information and Communication Technology, Le Quy Don Technical University, Hanoi, Vietnam; Hubei University, CHINA

## Abstract

System logs are widely used for monitoring system reliability and detecting abnormal behaviors in large-scale computing infrastructures. While deep learning has significantly advanced log anomaly detection, most existing methods are confined to in-domain scenarios, relying heavily on abundant system-specific historical data. Consequently, they struggle to monitor newly deployed systems where log data and anomaly labels are severely scarce. Achieving robust cross-system log anomaly detection remains a challenge due to inherent domain gaps, including completely different logging structures, continuously evolving templates and severe Out-Of-Vocabulary issues. To bridge these gaps, we propose MeCoLog, a Meta-Contrastive learning method for cross-system log anomaly detection. First, a Hybrid Parameter Embedding is introduced to encode type, value, and key information to handle diverse vocabularies. These embeddings are integrated into the model via a Gated Key Injection mechanism and Rotary Position Embedding. These allow the model to capture both event relevance and execution flow. Second, we design an Asymmetric Prototype Contrastive Learning objective. This method aligns normal behaviors across different systems while strictly isolating anomalies. Together, this design enables MeCoLog to achieve robust performance in few-shot scenarios. Extensive experiments on multiple public log datasets (HDFS, BGL, Thunderbird, Hadoop and AIT-LDS v2.0) demonstrate that MeCoLog consistently outperforms state-of-the-art baselines under few-shot cross-system settings, offering a robust and highly transferable solution for real-world log anomaly detection.

## Introduction

Modern large-scale computing infrastructures generate massive volumes of system logs. These infrastructures include cloud platforms, distributed storage systems, and microservice-based services. The logs capture runtime events, execution states, resource usage, warnings, and failures. They serve as critical evidence for system monitoring, fault diagnosis, and security analytics [1–4]. In cybersecurity management systems, log analysis plays a key role. Abnormal log patterns often provide early indicators of service disruption, misconfiguration, malicious activity and ongoing attacks [3,5,6]. Modern infrastructures continue to grow in scale and complexity. As a result, manual inspection of logs becomes impractical, making it increasingly challenging to effectively monitor and analyze log data for potential security threats. This trend has motivated the development of automated log anomaly detection techniques [3].

Early methods for log anomaly detection relied on handcrafted rules or statistical analysis of event frequencies [1,7]. More recently, deep learning approaches have advanced the field. These approaches learn sequence representations directly from log data. Sequence models such as LSTM and Transformer architectures capture temporal dependencies among log events [5,6,8]. Representation-learning methods employ pretrained language models and contextual embeddings [3,9]. These methods achieve strong performance within individual systems. However, their effectiveness often tends to diminish when applied to new environments [10,11]. Full retraining for every newly monitored system is often infeasible due to the prohibitive cost of collecting labeled anomalies. To address this, cross-system log anomaly detection aims to leverage knowledge extracted from previously observed environments to secure new ones. This approach enables rapid adaptation to target systems using only a minimal number of labeled samples [10,12,13]. This setting is practically important because target systems often provide only limited labeled data. Such efficiency is essential for defenders to maintain robust performance even under significant system shifts.

Cross-system log anomaly detection presents several challenges. First, anomalous samples remain scarce. A newly monitored target system may provide only a handful of labeled examples for calibration. Standard supervised methods [9] fail in this regime because they require large annotated datasets. Similarly, semi-supervised methods are sensitive to distributional drift and cannot effectively leverage the few available anomaly labels [11,14,15]. Therefore, rapid and data-efficient adaptation to new systems requires an inductive learning strategy optimized for few-shot generalization.

**Fig 1:**
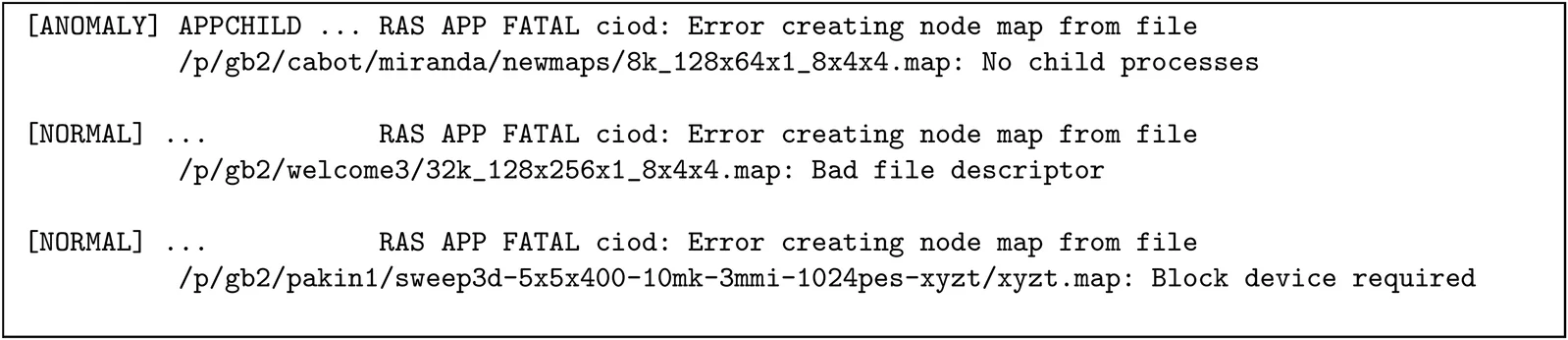
Drain output for BGL datasets. All three log lines share the same template, yet the anomaly signal is carried exclusively by the parameters (file path and error description).

Second, the domain gap across systems appears in both log structure and parameter semantics. Different systems use different vocabularies, template structures, logging styles, abbreviations, and parameter distributions [10–12]. As a result, even similar events, such as failed authentication or disk write errors, may be described with different words and patterns. Standard log parsers such as Drain [7] normalize logs by replacing variable fields with generic wildcards. Although this reduces vocabulary variation, it also removes parameter information and can create *Out-Of-Vocabulary* (OOV) issues when templates from a new system are encountered. This limits the transferability of methods that rely on system-specific token vocabularies. In addition, variable parameters often contain important anomaly evidence, such as abnormal resource values, unusual IP addresses, file paths, node identifiers, or system states [9,16]. These signals are often more system-independent than templates, making them especially useful for cross-system transfer. However, existing methods usually ignore this information or do not align it well across systems. Fig 1 shows examples where log lines share the same template, but the anomaly is only revealed by the parameter content.

Third, current methods lack an effective discriminative mechanism that accounts for the inherent distribution asymmetry between normal and anomalous log sequences. Normal operational behaviors usually exhibit stable, recurring patterns that are largely consistent across different systems [5,17]. In contrast, anomalies are fundamentally different. They arise from unpredictable sources such as misconfigurations, hardware faults, or cyber attacks. This makes them inherently diverse and structurally arbitrary. Existing detection objectives often overlook this geometric distinction. They either assume a single closed-loop boundary for all data or force diverse anomalies into a single artificial cluster.

Based on these fundamental challenges, this study aims to address the following three research questions:

- **RQ1** (Knowledge Transfer): How can a model effectively transfer diagnostic knowledge from a source system to a target system when only a minimal number of labeled samples are available?
- **RQ2** (Feature Extraction): How can semantic templates and heterogeneous log parameters be integrated into a unified representation that remains robust across diverse system environments?
- **RQ3** (Representation Learning): How can the model leverage the natural structure of log data to improve cross-system transferability?

To address the cross-system knowledge transfer challenge (RQ1), we propose MeCoLog (**Me**ta-**Co**ntrastive Learning for **Log** Anomaly Detection). This method formulates the detection task as an episodic few-shot learning problem. This approach addresses both label scarcity and domain gap by training the model on a source system and adapting it to a new target system using only a small labeled support set, without any retraining.

To optimize feature extraction from heterogeneous log data (RQ2), we integrate structured parameter information directly into log sequence embeddings through a Hybrid Parameters Embedding approach. This mechanism preserves the full semantic content of log messages by applying specialized encoding branches for different parameter types. These embeddings are then fused into the Transformer attention mechanism via our proposed Gated Key Injection (GKI) mechanism, allowing parameter context to selectively modulate inter-event relevance. Furthermore, relative sequential dependencies are captured through Rotary Position Embedding (RoPE) [18], ensuring the encoder remains sensitive to the execution flow rather than absolute event positions.

To address the challenge of representation learning (RQ3), we introduce an Asymmetric Prototype Contrastive Loss (APCL). This objective is based on the observation that normal behaviors usually reflect stable, system-agnostic operational states. Consequently, they serve as the primary anchor for knowledge transfer. In contrast, anomalous behaviors are far more heterogeneous and should not be forced into a single, artificial cluster. Our asymmetric design therefore encourages normal samples from diverse systems to form compact, transferable clusters that serve as a reliable reference in the target domain. In contrast, it allows anomalies to remain diverse and dispersed in the embedding space. Combined with metric-based meta-learning, this objective shapes a representation space. By leveraging the natural stability of normal data, it supports robust cross-system transfer under few-shot conditions.

The main contributions of this work are as follows:

- We propose **MeCoLog**, a meta-learning method that formulates cross-system log anomaly detection as an episodic few-shot problem. This enables rapid, training-free adaptation to new target systems using only a minimal labeled support set.
- We design a Hybrid Parameter Embedding that encodes log parameters in a domain-agnostic manner, resolving cross-system OOV issues. These features are integrated via a Gated Key Injection (GKI) mechanism and Rotary Position Embedding (RoPE), allowing the model to detect both semantic and sequential anomalies within a unified attention computation.
- We introduce an Asymmetric Prototype Contrastive Loss (APCL) that leverages the natural geometry of log data. By encouraging compact clusters for normal patterns and allowing diverse dispersion for anomalies, APCL yields a representation space that is both highly transferable and discriminative under few-shot conditions.
- We conduct comprehensive experiments on multiple real-world datasets, including HDFS, BGL, Thunderbird, and AIT-LDS. The results demonstrate that MeCoLog significantly outperforms state-of-the-art baselines in few-shot cross-system scenarios, maintaining high detection accuracy and operational efficiency.

The remainder of this paper is organized as follows: Section 2 reviews related work and provides motivation. Section 3 presents the MeCoLog method in detail. Section 4 reports experimental setup and results. Section 5 concludes the paper and discusses future directions.

## Background and motivation

### Single-system log anomaly detection

Log anomaly detection typically follows a four-stage pipeline: log collection, log parsing, feature extraction, and anomaly detection [2,3,19]. Raw log records encoding execution events are transformed by a log parser (e.g., Drain [7], Spell [20], or UniParser [21]) into structured templates by replacing variable fields with wildcards. The resulting template sequences are encoded into numerical representations, such as event count vectors or ordered key sequences grouped by windows [1,5], before being passed to the detection model. These models differ substantially in their supervision assumptions, spanning supervised approaches (e.g., LogRobust [9]), semi-supervised methods that model normal execution paths (e.g., DeepLog [5], LogAnomaly [6], LogBERT [8]), and unsupervised clustering techniques (e.g., LogCluster [22], NeuralLog [16]). Despite their efficacy, all three paradigms are fundamentally restricted to single-system settings and degrade substantially under cross-system deployment due to shifting logging behaviors [10–12].

To capture anomalies invisible to template-only configurations, a parallel line of work explicitly integrates parameter information into log representations. HitAnomaly [23] utilizes separate Transformer encoders for templates and parameters, while Chai et al. [24] preserve parameter semantics within a BERT framework. Similarly, LogFormer [25] injects character-level parameter encodings as scalar attention biases. Crucially, while these methods confirm the value of parameter-aware representations, they remain inherently tied to source-system vocabularies or apply biases uniformly without distinguishing template keywords from parameters. Furthermore, their multi-stage pipelines are not designed for the label-scarce, cross-system regime that MeCoLog targets.

### Cross-system log anomaly detection

Cross-system log anomaly detection aims to deploy models on unseen target domains with minimal supervision by leveraging knowledge from source systems [10,12,13]. Existing strategies explore adversarial domain adaptation to align global feature distributions (LogTransfer [10]), unsupervised nearest-neighbor scoring (Han et al. [12]), structural graph Transformers (LogGT [11]), and episodic meta-learning for rapid adaptation (MetaLog [13]).

**Fig 2:**
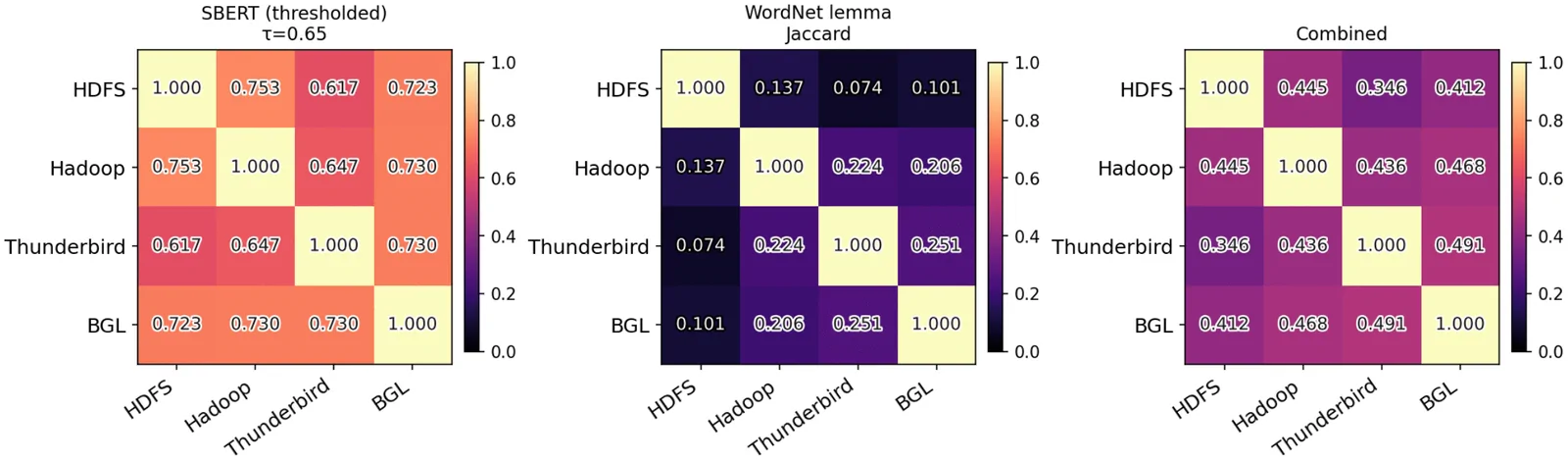
Semantic and Lexical Decomposition of Template Vocabularies. The figure compares cross-dataset similarity using three metrics: a) SBERT (thresholded, τ=0.65): Measures semantic similarity by mapping templates into a dense vector space to capture related technical terms. b) WordNet Lemma Jaccard: Evaluates strict lexical overlap based on normalized word lemmas, highlighting the lack of shared linguistic base words. c) Combined: An integrated view of both semantic and lexical proximity, illustrating the fundamental “vocabulary gap” across different system environments.

Despite these advancements, cross-system deployment faces a fundamental linguistic and structural gap driven by disparate logging libraries, template formats, and parameter distributions. Traditional parsing discards crucial system-agnostic operational semantics (e.g., numerical resource metrics or node identifiers) through wildcard substitution, eliminating anchors that could establish cross-system correspondence. Even operationally identical behaviors are expressed through entirely different structures. As quantified in Fig 2, while dense semantic encoders like SBERT [26] may suggest moderate proximity (0.62–0.75) via coarse geometric clustering of technical terms, strict lexical measures like the WordNet Lemma Jaccard similarity reveal near-total disjointedness (0.07–0.25). Models anchored to source-specific surface tokens lack the reliable lexical footing necessary to generalize, resulting in poor performance under domain shifts.

**Fig 3:**
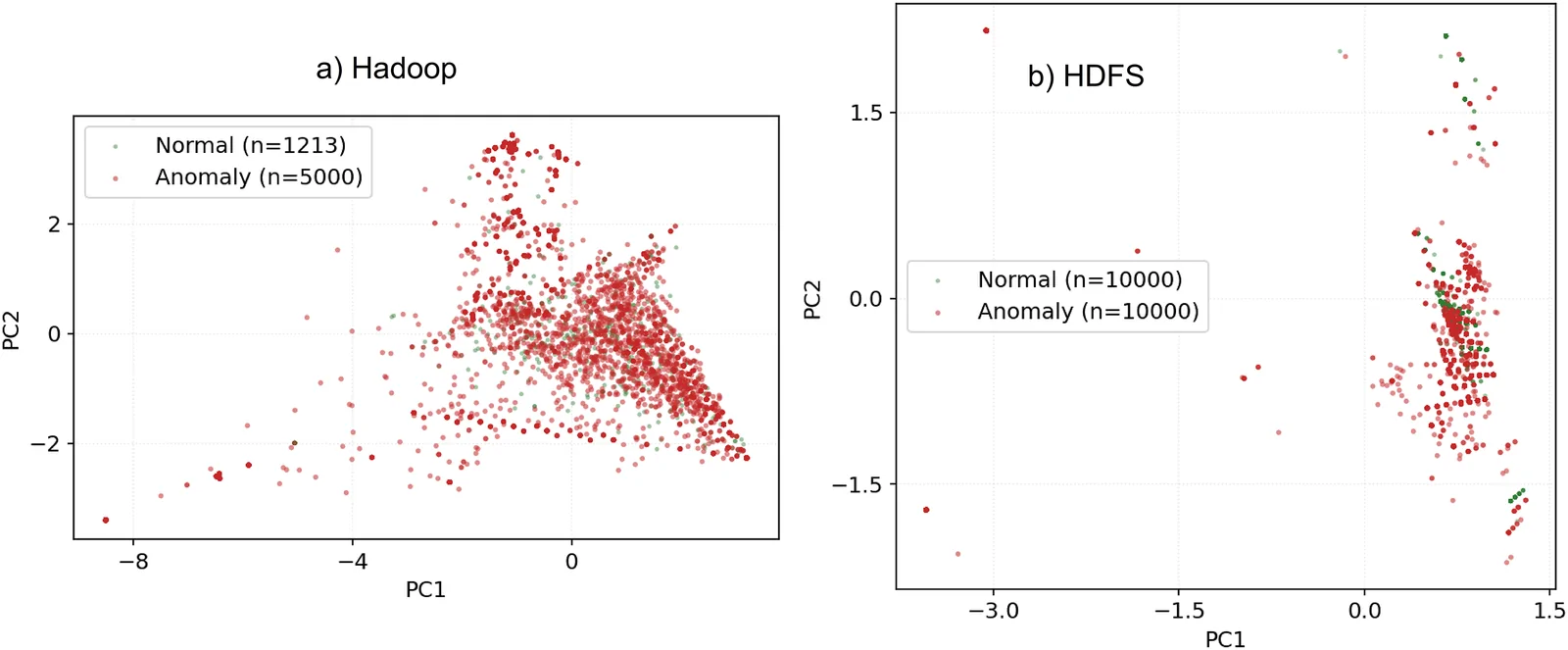
Dispersion of Raw data: Diversity and arbitrary shape of anomalies.

Furthermore, existing cross-system paradigms fail to account for the asymmetric geometry of normal versus anomalous behaviors under few-shot constraints. In real-world deployments, anomalies are severely underrepresented, often comprising less than one percent of log streams [3]. Consequently, a target support set of a few labeled instances cannot cover the full diversity of arbitrary system failures. As visualized in Fig 3, while normal behaviors form compact, stable clusters, anomalies exhibit a high degree of dispersion and unpredictable structural shapes. Contrastive objectives [14,27] that enforce uniform compactness across all classes are therefore ill-suited to this domain. While metric-based meta-learning networks [28,29] optimize for fast adaptation with sparse data, they must be coupled with an embedding space that respects this natural asymmetry. MeCoLog directly addresses these compounding limitations by fusing hybrid parameter encoding with an asymmetric contrastive learning objective within an episodic meta-learning framework.

## Methodology

### Overview

**Fig 4:**
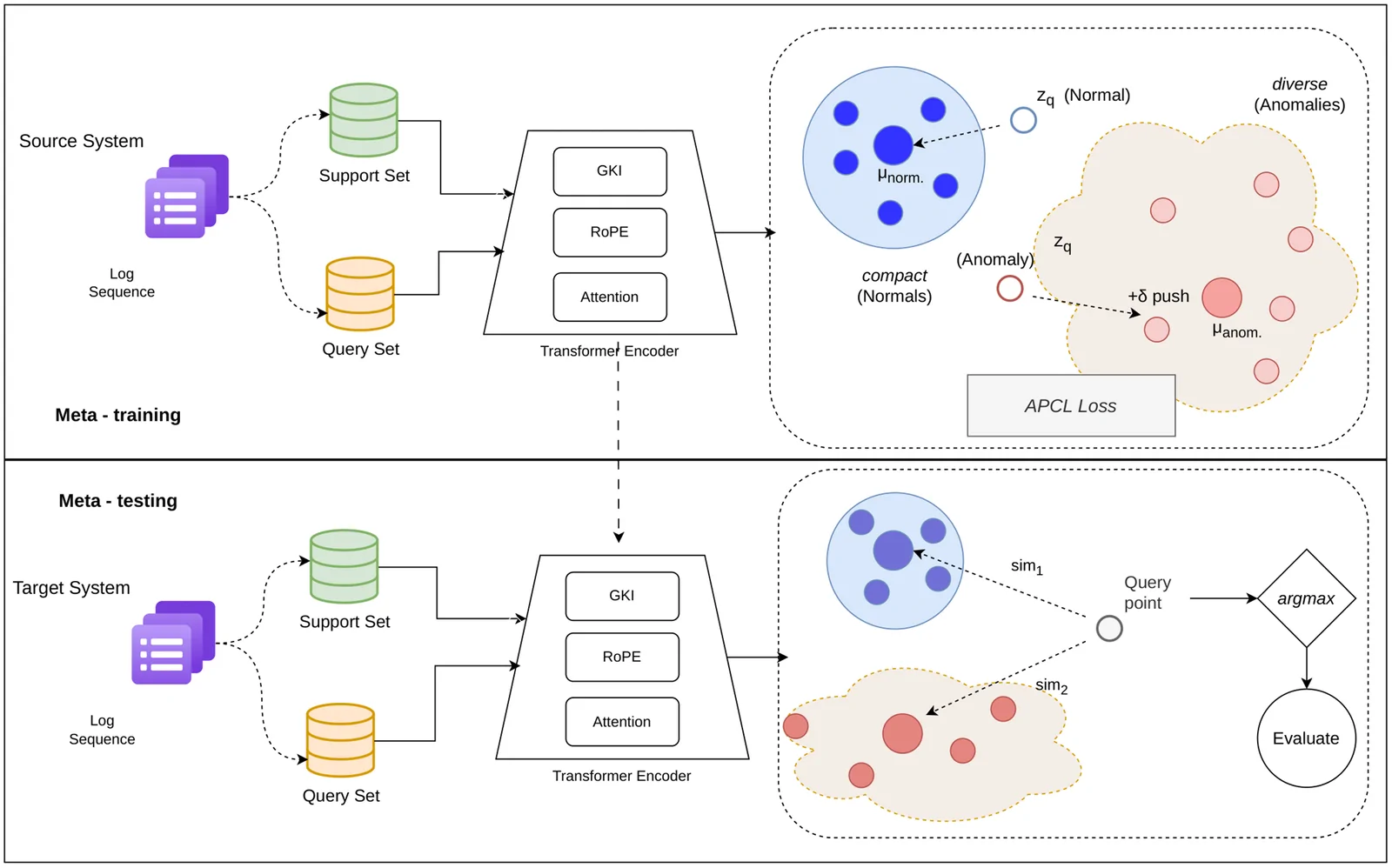
Overview of the MeCoLog method.

We propose MeCoLog, a metric-based meta-learning method for cross-system log anomaly detection. The method addresses the two principal challenges of cross-system deployment: label scarcity in the target system and the domain gap between heterogeneous logging environments. The overall pipeline proceeds as follows. Raw log sequences are first parsed into structured representations consisting of a template and a set of typed parameters. For each log event, type-specific parameter embeddings are constructed and aggregated into a single parameter embedding. Template and parameter vectors are then fed jointly into a Transformer encoder, where parameter information is injected into the attention mechanism through a GKI mechanism. The encoder produces a sequence-level embedding that captures both template semantics and operational parameter context. These embeddings are optimized during meta-training on source systems via episodic sampling and an APCL. For deployment, a small labeled support set is collected from the target system, while a query set from the test suite is used to assess model performance. Fig 4 provides a detailed overview of this pipeline.

### Parameter integration

**Fig 5:**
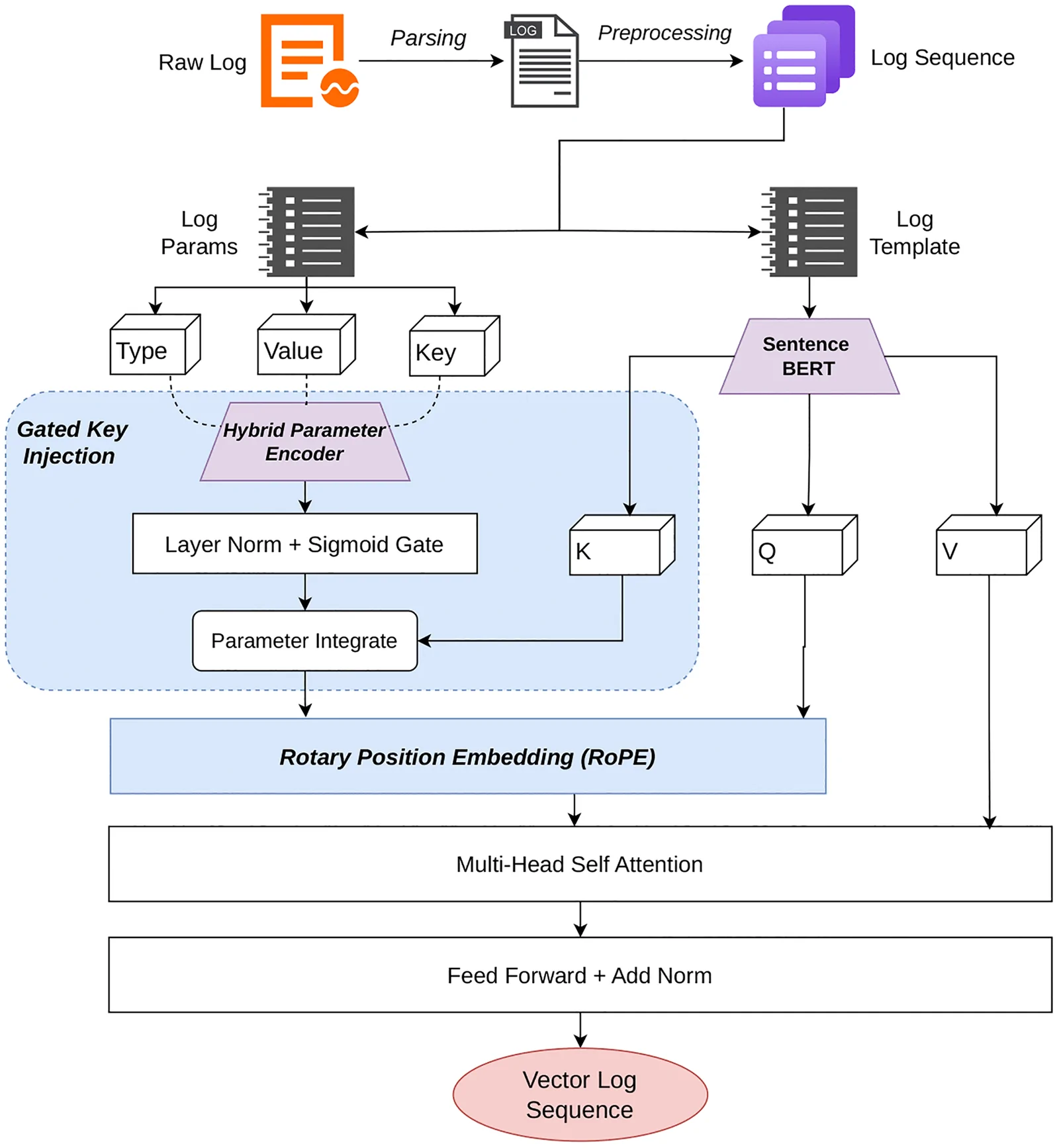
The Parameter-Integrated Transformer Block. The diagram illustrates our proposed *Hybrid Parameter Encoder* and the *GKI* process. These components, combined with the integrated *RoPE*, enable the model to modulate inter-event attention based on specific parameter values while preserving relative positional context across heterogeneous log sequences.

Standard log parsers replace variable parameters with wildcards, discarding critical operational context such as resource identifiers, numerical values, system states, and path information [7]. This also creates cross-system incompatibility because value vocabularies differ between systems. We address both issues by constructing type-specific parameter embeddings that encode operational semantics in a domain-agnostic manner. These embeddings are then injected into the Transformer encoder jointly with relative positional information. Fig 5 illustrates the overall architecture for this integration. It shows how the parameter vectors are fused with template embeddings and relative positional information within the Transformer encoder.

### Hybrid parameters embedding

For each log event, we extract parameters using regular expressions and represent them as tuples (k,v,τ), where *k* is the key, *v* is the value, and τ is the type. The type τ is inferred from predefined patterns and belongs to the set {NUM,IP,HEX,PATH,STR}. If a parameter appears in explicit key-value form (e.g., user = root), the key is extracted directly; otherwise, a positional key $k={POS}_{j}$ is assigned according to its order in the line. The set of positional keys is fixed and shared across all datasets.

**Fig 6:**
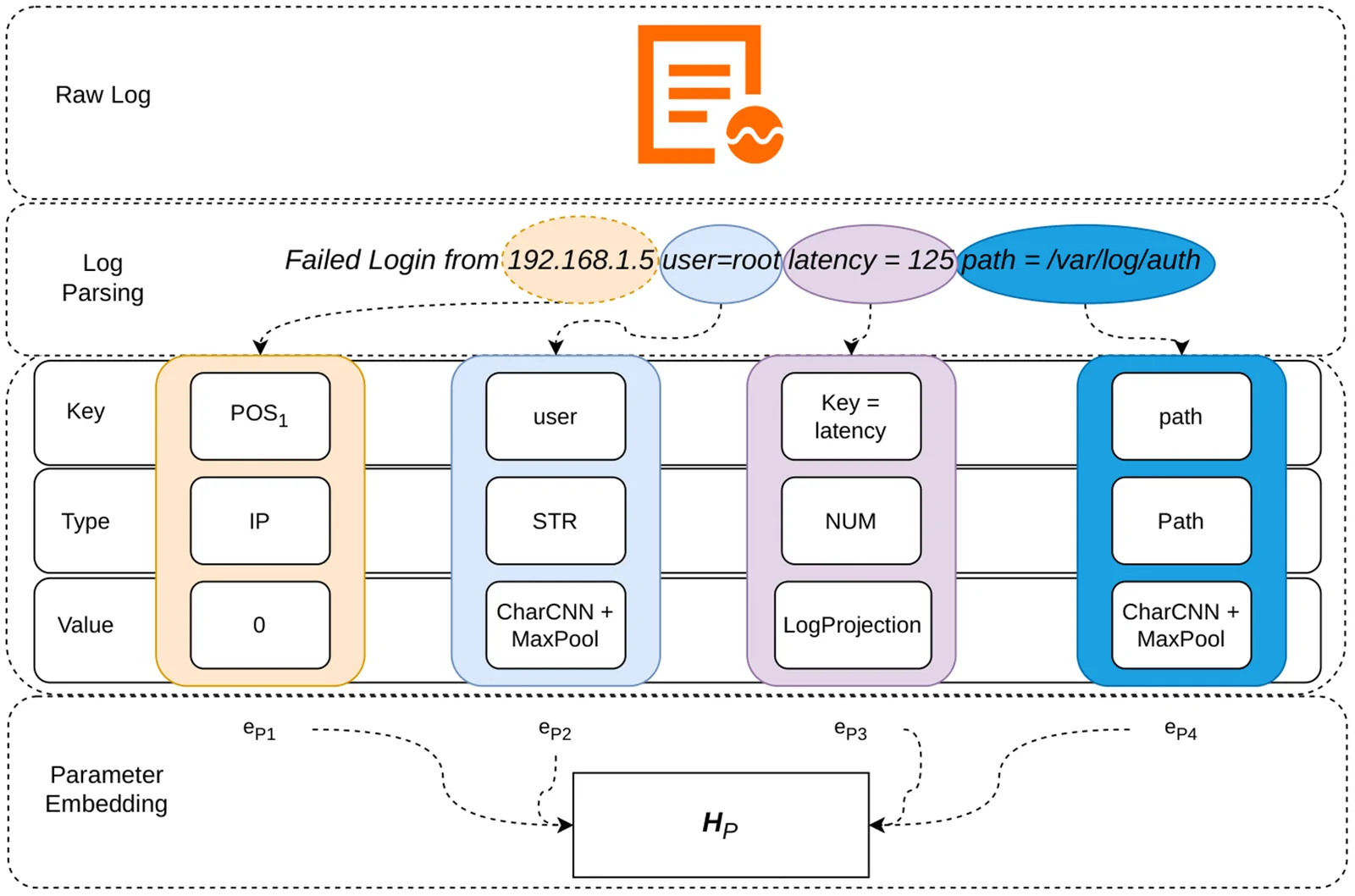
Specialized encoding pipelines for different parameter types.

Value embeddings are computed differently according to type to preserve operational semantics without relying on dataset-specific vocabularies. For categorical strings (τ∈{STR,PATH}), we apply a character-level CNN encoder: the value is tokenized into characters, embedded, passed through 1D convolution with ReLU activation, and max-pooled. For paths, a normalization step ${𝒩}_{path}(\cdot )$ preserves structural delimiters while replacing volatile numeric or hexadecimal substrings with shared placeholders (e.g., ⟨NUM⟩). Numerical values (τ=NUM) are projected using a log-scaled transformation that preserves magnitude without allowing large values to dominate. High-cardinality identifiers (τ∈{IP,HEX}) contribute only through their type embedding (${𝐞}_{val}=0$). IP addresses and hexadecimal tokens are instance-specific identifiers that vary arbitrarily across systems and log lines; encoding their values would introduce system-specific noise into the representation rather than transferable semantics. The specific encoding workflows for these diverse parameter types are illustrated in Fig 6. Formally:


$$\begin{array}{c} e_{val}(v,\tau )=\left\{ \begin{array}{cc} CharCNN(v), & \tau =STR,\\ CharCNN({𝒩}_{path}(v)), & \tau =PATH,\\ {𝐖}_{num}\cdot sgn(v)\ln(1+|v|)+{𝐛}_{num}, & \tau =NUM,\\ 0, & \tau \in \{IP,HEX\}. \end{array}\right. \end{array}$$ (1)

where ${𝐖}_{num}\in {\mathbb{R}}^{1\times d}$ and ${𝐛}_{num}\in {\mathbb{R}}^{d}$ are learnable parameters, and sgn(v) is the sign function that preserves the algebraic direction of the value.

MeCoLog employs an additive factorization strategy to construct parameter embeddings, utilizing learnable lookup tables for keys and types to ensure cross-system stability. Keys and types are embedded with small learnable lookup tables that depend only on explicit field names or positional identifiers. Unseen explicit keys at meta-testing are mapped to a shared [UNK_KEY] token, ensuring the key vocabulary remains stable across systems. Let ${𝐞}_{key}(k)\in {\mathbb{R}}^{d}$ and ${𝐞}_{type}(\tau )\in {\mathbb{R}}^{d}$ denote the embeddings for keys and types, respectively, retrieved from learnable lookup tables. The total parameter embedding ${𝐞}_{param}\in {\mathbb{R}}^{d}$ for a single (k,v,τ) tuple is formed through additive factorization:


$$\begin{array}{c} {𝐞}_{param}(k,v,\tau )={𝐞}_{key}(k)+{𝐞}_{type}(\tau )+{𝐞}_{val}(v,\tau ). \end{array}$$ (2)

This factorization lets key semantics, structural type, and value information contribute independently to the downstream attention computation. For an event with *M* parameters, the individual parameter embeddings are summed to obtain a single permutation-invariant parameter vector:


$$\begin{array}{c} {𝐡}_{P}^{\left(i\right)}=\sum_{m=1}^{M}{𝐞}_{param}(k_{m},v_{m},\tau_{m}). \end{array}$$ (3)

### Integrated parameter and sequence information

Each log event therefore carries two representations: a template semantic embedding ${𝐡}_{T}^{\left(i\right)}$ produced by Sentence-BERT [26], and its parameter vector ${𝐡}_{P}^{\left(i\right)}$. A sequence of *L* events, prepended with a learnable [CLS] token, yields template and parameter matrices ${𝐇}_{T},{𝐇}_{P}\in {\mathbb{R}}^{(L+1)\times d}$. The SBERT model is kept frozen during meta-training to serve as a robust and general-purpose feature extractor. This design follows the large-small model collaboration paradigm [30], where a large pretrained model provides semantic grounding while a lightweight module handles task-specific adaptation. This prevents the model from overfitting to the specific vocabulary of the source systems and ensures that the learned template embeddings remain transferable across heterogeneous logging environments.

MeCoLog modulates inter-event relevance by injecting the parameter matrix ${𝐇}_{P}$ directly into the key projection of each Transformer self-attention layer. This design is based on the role of the key in determining which events receive more attention, while the query and value are responsible for information retrieval. By augmenting the key with parameter embeddings, the model lets the operational context of each log event adjust its influence on the sequence representation. The core semantic information extracted per event remains unaltered. Concretely, for each attention head *h*, the query and value projections are derived from the template embeddings in the standard manner, while the key is computed as:


$$\begin{array}{c} {𝐊}_{content}^{h}={𝐇}_{T}{𝐖}_{K}^{h}+\sigma ({𝐇}_{P}{𝐖}_{gate}^{h})⊙LN({𝐇}_{P}{𝐖}_{P}^{h}), \end{array}$$ (4)

where ${𝐇}_{T},{𝐇}_{P}$ are the template and parameter matrices, ${𝐖}_{K}^{h}$ is the standard key projection matrix, ${𝐖}_{gate}^{h}$ and ${𝐖}_{P}^{h}$ are learnable projection matrices for the gating and parameter branches respectively, σ(·) denotes the sigmoid function, LN denotes layer normalization, and ⊙ is the element-wise product. The sigmoid gate selectively scales the contribution of each parameter embedding before it enters the key, suppressing high-cardinality noise while allowing semantically meaningful parameters to modulate inter-event similarity.

MeCoLog incorporates RoPE [18] to capture the relative temporal dependencies between log events, which are more critical for anomaly detection than absolute positioning. Standard positional encodings often conflate positional and semantic signals by adding fixed offsets, making attention scores a function of absolute indices rather than relative distances. This is insufficient for log sequences, where anomalous executions typically manifest as violated ordering constraints or unexpected temporal gaps, signals that are defined by the distance between events rather than their specific positions in a window. By applying RoPE directly to the query and augmented key projections, the model encodes position by rotating vectors in a multi-dimensional complex space. This ensures that the dot product between a query at position *m* and a key at position *n* depends solely on the relative offset (m−n), allowing the Transformer to focus on the sequential flow of the system state.


$$\begin{array}{c} \langle {ℛ}_{m}𝐪,\;{ℛ}_{n}𝐤\rangle =f\;\left(𝐪,𝐤,\;m-n\right), \end{array}$$ (5)

${ℛ}_{m},{ℛ}_{n}\in {\mathbb{R}}^{d_{h}\times d_{h}}$ are the block-diagonal rotation matrices applied at positions *m* and *n* respectively, and f(𝐪,𝐤,m−n) denotes the resulting attention score, which depends only on the relative offset (m−n) rather than absolute positions. This means attention scores between any two log events encode how far apart they are in the execution flow, independent of where the window starts. As a desirable consequence, attention naturally decays with increasing event distance [18]. Nearby events receive higher influence, while long-range dependencies can still be captured when necessary.

The final query and key projections entering the attention computation are:


$$\begin{array}{c} {𝐐}^{h}=ℛ\;\left({𝐇}_{T}{𝐖}_{Q}^{h}\right), \end{array}$$ (6)

$$\begin{array}{c} {𝐊}^{h}=ℛ\;\left({𝐊}_{content}^{h}\right), \end{array}$$ (7)

where ℛ(·) denotes the position-wise RoPE rotation applied element-wise to each row according to the event’s index in the sequence. The attention score between events at positions *m* and *n* then becomes:


$$\begin{array}{c} A_{mn}^{h}=\frac{{\left({ℛ}_{m}{𝐪}_{m}^{h}\right)}^{⊤}\left({ℛ}_{n}{𝐤}_{n}^{h}\right)}{\sqrt{d_{k}}}, \end{array}$$ (8)

where $d_{k}=d_{h}$ is the key dimensionality used as a scaling factor to stabilize gradients, and the score depends on both the semantic and parameter content of the two events (encoded in ${𝐪}_{m}^{h}$ and ${𝐤}_{n}^{h}$ via GKI) and their relative position m−n (encoded by the rotation).

The integration of GKI and RoPE provides both content and order information. While GKI enriches the semantics of each key by fusing template evidence with parameter context, RoPE applies a position-dependent orthogonal transformation to these enriched vectors. Because RoPE is norm-preserving, the magnitude-based gating effects of GKI remain invariant under rotation. The integrity of parameter-driven attention modulation is therefore maintained. This composition allows the model to simultaneously evaluate the operational significance of an event (via GKI) and its relative temporal distance (via RoPE) without mutual interference.

Together, GKI encodes the operational context of each individual event, while RoPE makes inter-event attention sensitive to sequential distance. This enables the encoder to detect both *semantic* anomalies (wrong parameter values) and *sequential* anomalies (correct events in a wrong order) within the same unified attention computation.

The full encoder stack aggregates global sequence context with both parameter awareness and relative position sensitivity. The final sequence embedding ${𝐳}_{seq}$ is taken from the [CLS] output position and passed to the APCL objective during meta-training and to the prototype inference procedure at meta-testing.

### Episodic meta-learning

We formulate cross-system log anomaly detection as a few-shot metric-learning problem under the episodic meta-learning paradigm [28,29]. Training tasks are sampled from a distribution p(𝒯) of episodic tasks constructed from the source system. Each task ${𝒯}_{i}$ is a **N-way *K*-shot** episode: N classes (normal and one or many anomaly classes), each represented by *K* support samples. Concretely, each episode consists of:

- A **support set** $𝒮={𝒮}_{norm}\cup {𝒮}_{anom}$, where $|{𝒮}_{norm}|=|{𝒮}_{anom}|=K$. This simulates the few-shot calibration available at deployment. ${𝒮}_{norm}$ and ${𝒮}_{anom}$ are the support sets of normal and anomalous sequences respectively
- A **query set** 𝒬, drawn from the same system but disjoint from 𝒮, used to compute the training loss.

The value of *K* is varied across experiments to assess sensitivity to support set size. During meta-training the model parameters ϕ are updated so that performance on 𝒬 improves after conditioning on 𝒮. During meta-testing on a new target system, only a small labeled support set is available; the model reuses the transferable embedding learned from source systems and calibrates the detection boundary on the fly without any retraining.

The episodic procedure directly mirrors the target deployment scenario. By training on diverse episodic tasks sampled from the source system, the model learns a general adaptation strategy rather than source-specific patterns. This means no distributional alignment between source and target systems is assumed or required. The model learns how to adapt, not what the target looks like.

### Asymmetric prototype contrastive loss

Standard supervised contrastive objectives treat all classes symmetrically, which is appropriate only when all classes are equally compact. Log anomaly detection violates this assumption: normal behaviors are semantically stable and form compact clusters, whereas anomalies arise from diverse, unpredictable causes. A symmetric loss imposes a false structural equivalence on these distinct data geometries.

We propose a loss that reflects this asymmetry directly. Normal queries use standard prototype cross-entropy to tighten the normal cluster. Anomaly queries use a margin-augmented variant that concentrates gradients on “hard” anomalies, namely those closest to the normal prototype. It does not impose a compactness constraint on anomaly embeddings. Our Asymmetric Prototype Contrastive Loss (APCL) inflates the normal prototype similarity for anomaly queries using an adaptive margin $\delta (z_{q})$ during training.

For each episode, both class prototypes are computed from the support set as equations below. All embeddings are L2-normalized onto the unit hypersphere before computing similarities:


$$\begin{array}{c} \mu_{norm}=\frac{1}{\left|{𝒮}_{norm}\right|}\sum_{x_{s}\in {𝒮}_{norm}}f_{\phi }(x_{s}), \end{array}$$ (9)

$$\begin{array}{c} \mu_{anom}=\frac{1}{\left|{𝒮}_{anom}\right|}\sum_{x_{a}\in {𝒮}_{anom}}f_{\phi }(x_{a}). \end{array}$$ (10)

where $f_{\phi }(\cdot )$ is the sequence encoder parameterized by ϕ, and $\mu_{norm},\mu_{anom}\in {\mathbb{R}}^{d}$ are the resulting class prototypes.

To separate anomalies from normal samples, we propose the Adaptive Margin Mechanism, which dynamically penalizes anomalies residing near the normal cluster. The difficulty of an anomaly query $z_{a}$ is quantified by its proximity to $\mu_{norm}$:


$$\begin{array}{c} \delta (z_{a})=\max(0,z_{a}\cdot \mu_{norm}) \end{array}$$ (11)

This formulation ensures that the margin grows proportionally to the proximity for hard anomalies, while remaining zero for anomalies already positioned in the opposite hemisphere. For a query sample $z_{q}$ with label $y_{q}\in \{normal,abnormal\}$, let $s(z_{q},\mu )$ denote the scaled cosine similarity $z_{q}\cdot \mu /\tau$. The margin-adjusted similarity to the normal prototype is defined as:


$$\begin{array}{c} \hat{s}(z_{q},\mu_{norm})=\left\{ \begin{array}{cc} (z_{q}\cdot \mu_{norm}+\delta (z_{q}))/\tau & \text{if }y_{q}=abnormal\\ (z_{q}\cdot \mu_{norm})/\tau & \text{if }y_{q}=normal \end{array}\right. \end{array}$$ (12)

The APCL loss treats the normal and anomalous queries asymmetrically by inflating the denominator for anomalies. The unified objective for a single query $z_{q}$ is expressed as:


$$\begin{array}{c} {ℒ}_{APCL}(z_{q},y_{q})=-\log\frac{\exp(s(z_{q},\mu y_{q}))}{\exp(\hat{s}(z_{q},\mu_{norm}))+\exp(s(z_{q},\mu_{anom}))} \end{array}$$ (13)

Finally, the total loss for a training episode is the average over the query set 𝒬:


$$\begin{array}{c} ℒ=\frac{1}{\left|𝒬\right|}\sum_{z_{q}\in 𝒬}{ℒ}_{APCL}(z_{q},y_{q}) \end{array}$$ (14)

The APCL design shapes the embedding space by encouraging normal samples to form a compact cluster while allowing anomalies to remain diverse across different system contexts. Anomaly queries are attracted toward an episode-local anomaly prototype through the numerator of Eq. 13. However, APCL removes the symmetric same-class pairwise compaction of standard contrastive objectives, and the anomaly prototype is recomputed per episode from varying anomaly compositions. No fixed global anomaly center therefore exists, and the adaptive margin concentrates the gradient on separating hard anomalies from the normal prototype rather than on compressing the anomaly class. This design links training more closely to inference, since the adaptive margin $\delta (z_{a})=\max(0,1-\text{Score}(z_{a}))$ assigns larger penalties to anomalies with higher inference risk. As a result, training focuses more on hard anomalies, namely those with low $\text{Score}(z_{a})=1-z_{a}\cdot \mu_{norm}$, which are more likely to be confused with normal samples at test time. This helps make the inference rule $\hat{y}(x_{q})=\text{arg}\max_{c}f_{\phi }(x_{q})\cdot \mu_{c}$ more robust, without requiring manual threshold tuning or F1-based grid search.

## Experiments

We conduct extensive experiments to evaluate the effectiveness and efficiency of MeCoLog. Our evaluation focuses on the following primary objectives:

- **Cross-system Performance Analysis.** We evaluate MeCoLog in few-shot cross-system anomaly detection scenarios. This analysis compares our method against state-of-the-art single-system and cross-system baselines, with a specific focus on its ability to capture diverse characteristics in datasets containing multiple distinct attack categories, such as AIT-LDS v2.0.
- **Ablation Study.** We perform a comprehensive ablation study to quantify the individual contributions of our core design elements: the Hybrid Parameters Embedding, the GKI mechanism and the APCL.
- **Sensitivity and Efficiency Evaluation.** We investigate the model’s sensitivity to key hyperparameters, specifically the temperature scale τ. Furthermore, we assess the operational efficiency of MeCoLog by measuring training convergence rates, inference latency, and the total model parameter count to demonstrate its suitability for real-time monitoring.

### Dataset

We evaluate the performance of MeCoLog using four widely recognized benchmark datasets: HDFS [1], BGL [31], Thunderbird [31], and Hadoop [22]. Each dataset comprises raw log lines labeled as either normal or anomalous. We process all datasets through a standardized pipeline of log parsing, template extraction, and sequence construction to ensure a fair and consistent comparison across all evaluated methods. For the HDFS dataset, we employ session-based grouping via BlockID, whereas Hadoop, BGL and Thunderbird are processed using a sliding window approach with a window size and step size both set to 20. Each system is split into training and testing partitions before any cross-domain experiment, with parameter vocabularies fit on the training partition only. All datasets use an 80/20 train/test split. For BGL and Thunderbird, a chronological split is used to preserve temporal ordering. For HDFS and Hadoop, the split is performed randomly at the session/block level. For cross-domain evaluation, MeCoLog meta-trains on the entire source training partition and evaluates exclusively on the target test partition. The few-shot support set at inference is drawn from the target test pool. Source test data and target training data are never used for model optimization.

The preprocessing details and statistics of HDFS, BGL, Hadoop and Thunderbird are summarized in Table 1. Besides sequence statistics, the table reports three structural characteristics: *Unique Templates*, the number of distinct log event types after parsing; *Template Vocab*, the number of unique tokens across all templates, reflecting lexical diversity; and *Effective Templates*, which measures the effective diversity of template usage by accounting for their frequency distribution. Unlike raw template count, this metric better captures structural complexity. Together, these characteristics quantify the structural gap between source and target systems, providing insight into the challenges of cross-system transfer.

**Table 1:** Statistical summary of benchmark datasets.

Dataset	Total Events	Grouping Strategy	Average Length	Normal Sequences	Abnormal Sequences	Unique Template	Template Vocab	Effective Template
HDFS	11,175,629	Session	19.4	558,223	16,838	48	117	8.5
BGL	4,713,493	Sliding	20.0	215,418	20,256	422	740	19.0
Thunderbird	2,101,180	Sliding	20.0	102,376	2,683	949	5,117	89.8
Hadoop	180,340	Sliding	20.0	1,213	7,804	213	598	31.5

To further assess the model’s robustness against diverse failure modes, we evaluate MeCoLog on the AIT Log Data Set v2.0 [32], which aggregates logs from eight different enterprises and covers various cyber-attack categories. In this study, we focus specifically on the access.log files from two enterprises, russellmitchell and santos, to examine the model’s performance in real-world server environments. AIT-LDS v2.0 uses a stratified 65/35 split to ensure that minority attack categories are represented in both partitions.

For sequence construction of AIT-LDS v2.0, we employ a sliding window approach with a window size of 20 and a smaller step size of 4 to ensure high coverage of sequential behaviors. We adopt a hybrid labeling strategy to categorize these sequences: generally, a sequence is labeled according to the majority attack type present within the window. However, to prioritize the detection of critical threats, any sequence containing at least one log line associated with severe attacks, such as webshell upload or privilege escalation, is automatically flagged as an anomaly of that specific type, regardless of the distribution of other labels. The severity override changes the fine-grained label of only 44 of 4,489 windows (0.98%). Crucially, all experiments on this dataset are formulated as binary anomaly detection, meaning each window is ultimately classified as either normal or anomalous. The fine-grained attack-type annotations are used solely for dataset characterization (Table 2) rather than prediction targets. Consequently, the reported metrics are identical under both the hybrid rule and a pure majority-vote rule. This override reflects security monitoring practice, where a window containing a critical event must not be labeled normal by majority, rather than a performance consideration.

**Table 2:** Detailed class distribution of the AIT-LDS v2.0 dataset, sorted by attack frequency.

Class	# Sequences	
	Russell-Mitchell	Santos
Normal	203	403
*Attacks:*		
Dirb	1,116	1,116
Wpscan	794	819
Escalate	1	10
Webshell Upload	6	6
Service Scan	6	5
Webshell Cmd	2	2
**Total**	**2,128**	**2,361**

For AIT-LDS v2.0, the support and query partitions are disjoint by construction, with the query set defined as the target test pool excluding the sampled support windows. For all other datasets, the step size equals the window size, so windows are non-overlapping. Combined with the cross-enterprise isolation between source training and target evaluation, no window is shared across the training, support, and query partitions.

During episodic meta-training, especially for multi-attack datasets like AIT-LDS v2.0, each episode is formed by sampling normal sequences and a diverse set of anomalous sequences spanning different attack categories. While the normal prototype ($\mu_{norm}$) is computed to form a compact cluster, the anomalous instances are treated via an asymmetric objective.

### Implementation details

Regarding the MeCoLog implementation, we use a parameter-aware Transformer encoder over fixed, precomputed 768-dimensional template embeddings. The architecture employs 1 encoder layer with 8 attention heads, RoPE positional encoding, and a GELU feed-forward sublayer of size 3072. Log-specific attributes are encoded by a type-aware parameter encoder and injected into attention through gated additive key refinement before the softmax layer.

For meta-training, we adopt episodic learning with a default 20-shot per class support set (normal and anomalous) and 10 query samples per class per episode. The APCL objective is optimized with a temperature τ=0.07 using Adam (learning rate 1e−5, weight decay 0) for 2000 default episodes. During inference, target domain test samples are scored via prototype-based comparison against the normal and anomalous support prototypes.

Log parsing is executed using Drain (similarity threshold 0.5, depth 4), with frozen template embeddings generated by the SBERT distilbert-base-nli-mean-tokens variant via mean pooling. All reported results are averaged over 5 independent runs. To ensure a fair comparison, the train/test data partition remains fixed under a single seed, while model initialization, episodic sampling, and meta-test support/query partitioning are varied across runs using five distinct seeds applied identically to all baselines. The source code is archived on Zenodo at https://doi.org/10.5281/zenodo.20487208. All experiments are conducted on an Ubuntu 22.04.3 LTS environment equipped with an Intel Xeon Gold 6240 CPU, an NVIDIA Quadro RTX 5000 GPU (16GB VRAM), and GPU Driver version 550.120.

### Baselines

To evaluate the effectiveness of MeCoLog, we compare it against two distinct groups of baseline models, ranging from classical single-system architectures to state-of-the-art cross-system methods.

*Single-System Log Anomaly Detection Baselines:* These models serve as benchmarks for assessing the fundamental discriminative power of log representations within a single domain:

- **DeepLog** [5]: A semi-supervised method using LSTMs to model system execution sequences as a language modeling task, flagging unexpected log key transitions.
- **LogAnomaly** [6]: An extension incorporating Word2Vec semantics to simultaneously capture sequential order and quantitative count-based deviations.
- **LogRobust** [9]: A supervised Bi-LSTM with attention engineered to handle template instability and noisy labels in real-world logs.

*Cross-System and Few-Shot Learning Baselines:* These methods explicitly address the challenges of domain shift and label scarcity in target systems:

- **LogTransfer** [10]: A supervised transfer learning approach that pre-trains an encoder on source data and fine-tunes the classifier on the target system.
- **MetaLog** [13]: A cross-system framework using a meta-learning strategy over source tasks to enable rapid target adaptation given a few-shot support set.
- **LogTAD** [12]: An unsupervised domain adaptation method that learns a unified representation space and measures distance from a global normal distribution.
- **LogFormer** [25]: A pre-training and adapter-based Transformer with character-level parameter encodings, whose two-stage pipeline requires labeled data from both domains.

All baselines utilize their originally designed feature representations and published hyperparameter configurations, as reliable re-tuning is impractical given the strict few-shot budget of the target system. The target few-shot support set (fixed at *K* = 20 per class, sharing identical query splits with MeCoLog) is applied via each baseline’s native adaptation mechanism. For semi-supervised models (DeepLog, LogAnomaly), which lack mechanisms to train on anomalies, support anomalies are used solely to calibrate the decision threshold. For supervised baselines (LogRobust, LogTransfer), the support samples are integrated into their respective training or fine-tuning procedures to ensure a consistent target-label budget across all evaluated methods.

### Evaluation metrics

Log anomaly detection is inherently class-imbalanced, since anomalous sequences constitute only a small fraction of the total data. Relying solely on accuracy can therefore be misleading. We therefore evaluate MeCoLog using the following comprehensive suite of metrics:

- Precision, Recall, and F1-score: We report the macro-averaged Precision and Recall to assess the model’s accuracy and completeness in identifying anomalies. The F1-score, calculated as the harmonic mean of Precision and Recall, serves as our primary metric for performance comparison.
- ROC-AUC: We include the Area Under the Receiver Operating Characteristic curve to measure the model’s ability to distinguish between normal and anomalous classes across various decision boundaries.

### Results and discussion

We evaluate the cross-system adaptability of MeCoLog through one-to-one (1→1) transfer experiments across all six representative transfer pairs within the BGL, HDFS, Thunderbird and Hadoop benchmark suite. As detailed in Table 3, our evaluation focuses on pairs with shared architectural characteristics. These include the distributed system environments of HDFS ↔ Hadoop and the supercomputing contexts of BGL ↔ Thunderbird. We also include a heterogeneous mixture pair (HDFS ↔ BGL) to test the model’s robustness under significant domain shifts. This structured comparison allows us to assess whether semantic and structural patterns learned from a source system can be effectively projected onto a previously unseen target environment with minimal supervision.

**Table 3:** Cross-system log anomaly detection performance across six one-to-one transfer pairs on four datasets (HDFS, BGL, Thunderbird and Hadoop). Each group reports AUC, Precision, Recall, and F1-score for eight methods under a 20-shot support set.

Scenario	Metric	DeepLog	LogAnomaly	LogRobust	LogTAD	MetaLog	LogTransfer	LogFormer	MeCoLog
HDFS → Hadoop	AUC	0.740	0.680	0.850	0.882	0.736	0.843	0.651	**0.934**
	Precision	0.867	0.667	0.810	0.825	0.867	0.915	0.540	**0.968**
	Recall	0.879	**1.000**	0.790	0.880	**1.000**	0.824	**1.000**	0.901
	F1	0.873	0.800	0.800	0.852	**0.929**	0.867	0.701	0.871
Hadoop → HDFS	AUC	0.815	0.815	0.879	0.785	**0.890**	0.861	0.873	**0.890**
	Precision	0.903	0.903	**1.000**	0.720	0.854	0.816	0.741	0.840
	Recall	0.652	0.752	0.367	0.755	0.792	0.749	**1.000**	**1.000**
	F1	0.757	0.821	0.537	0.737	0.822	0.781	0.851	**0.913**
Thunderbird → BGL	AUC	0.750	0.620	0.825	0.846	0.589	0.692	0.933	**0.949**
	Precision	0.650	0.486	**0.915**	0.725	0.835	0.820	0.805	0.841
	Recall	0.720	**1.000**	0.780	0.795	0.786	0.775	0.912	0.937
	F1	0.683	0.654	0.842	0.758	0.810	0.797	0.855	**0.886**
BGL → Thunderbird	AUC	0.614	0.648	0.845	0.804	0.638	0.689	0.832	**0.866**
	Precision	0.625	0.455	**0.880**	0.725	0.842	0.785	0.810	0.837
	Recall	0.794	**1.000**	0.750	0.795	0.761	0.694	0.798	0.928
	F1	0.700	0.625	0.810	0.758	0.799	0.737	0.804	**0.880**
HDFS → BGL	AUC	0.865	0.640	0.910	0.850	0.672	0.880	0.868	**0.954**
	Precision	0.715	0.559	**0.946**	0.785	0.722	0.744	0.820	0.850
	Recall	0.899	0.910	0.730	0.812	0.823	0.767	**0.950**	0.934
	F1	0.796	0.693	0.825	0.798	0.769	0.756	0.883	**0.890**
BGL → HDFS	AUC	0.859	0.829	0.853	0.815	0.739	0.648	0.892	**0.963**
	Precision	0.860	0.527	**1.000**	0.765	0.312	0.986	0.760	0.894
	Recall	0.699	0.681	0.367	0.720	0.208	0.515	**0.867**	0.767
	F1	0.771	0.594	0.537	0.742	0.250	0.677	**0.909**	0.826

**Fig 7:**
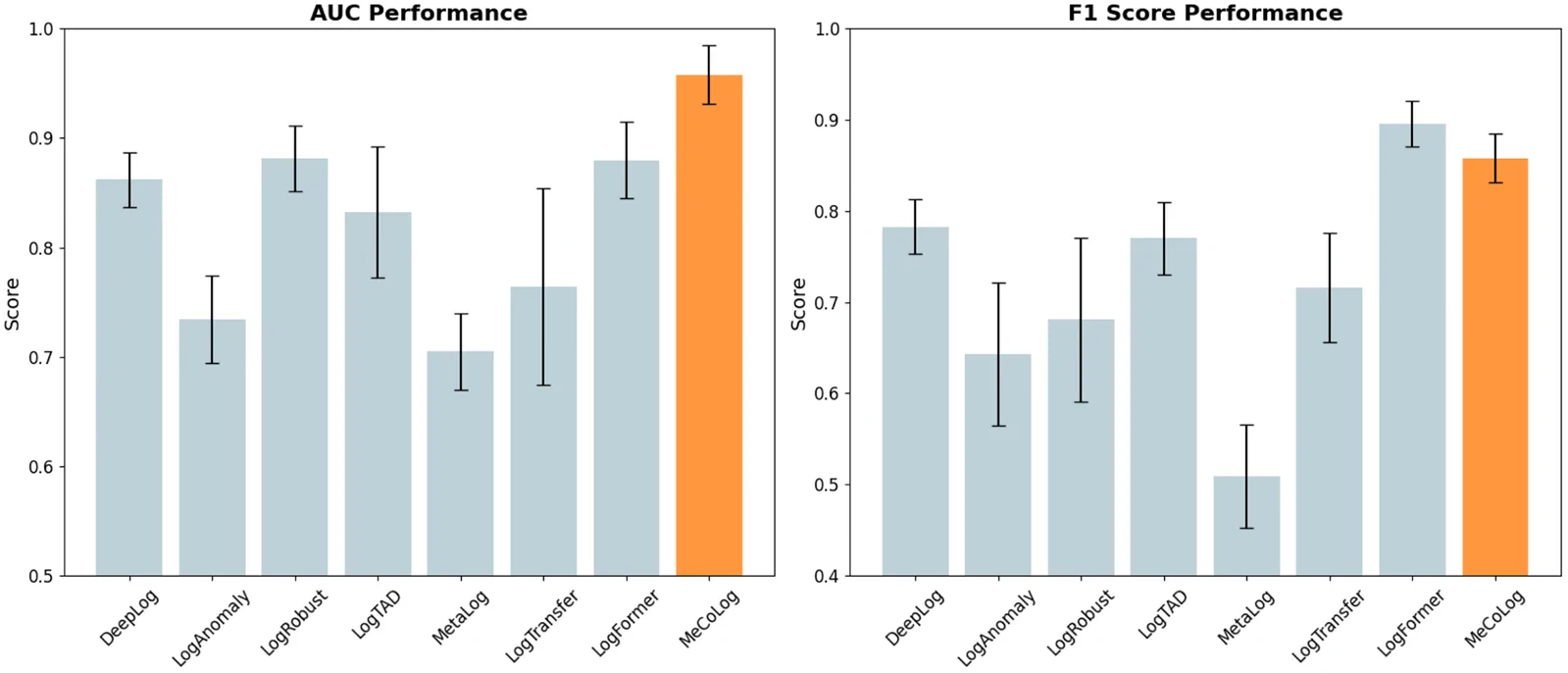
Error bar plot for the HDFS-to-BGL transfer scenario.

Table 3 presents the performance of MeCoLog compared to various baselines across six different cross-system transfer pairs. Overall, MeCoLog demonstrates superior adaptability and robustness, consistently achieving the highest F1-score and AUC in the majority of scenarios. In “homogeneous” transfer tasks where systems share similar architectures, such as HDFS ↔ Hadoop or BGL ↔ Thunderbird, MeCoLog maintains high precision and recall. It often surpasses strong baselines such as MetaLog and LogRobust. This indicates that our model effectively captures the underlying semantic and structural invariants common to similar system types. The HDFS → BGL and BGL → HDFS tasks involve significant domain shifts between different system categories. In these tasks, MeCoLog clearly outperforms the other methods in AUC, reaching 0.954 and 0.963 respectively. This confirms that the integration of GKI and APCL allows the model to generalize well even when the target system’s vocabulary and event distributions differ substantially from the source. Fig 7 shows the standard deviation of performance across multiple runs in the HDFS-to-BGL scenario, indicating the stability of the proposed MeCoLog method.

In several transfer scenarios, MeCoLog achieves the highest AUC while not leading on F1-score. This stems directly from the inference mechanism: MeCoLog assigns each query to its nearest prototype without any tunable decision threshold. Because anomalies in the target system are diverse and dispersed, some anomalous queries lie far from the anomaly prototype computed from the small support set and are assigned to the normal class. This lowers recall while precision remains high, producing a conservative operating point. AUC, as a threshold-free metric, is unaffected by this fixed operating point and more faithfully captures the model’s discriminative capability across all operating points. It is therefore our primary evaluation criterion.

To assess the risk of negative transfer arising from different logging semantics, we observe that the most prominent pattern is the correlation between cross-system semantic distance and the transfer performance gap. Specifically, when the combined similarity falls below a critical threshold, MetaLog suffers from severe representation collapse, whereas MeCoLog exhibits distance-agnostic robustness. The source domain complexity also shows a clear trend. High source diversity exacerbates embedding space dilution in traditional methods, whereas MeCoLog abstracts it into generalized knowledge. This robustness is attributable to SBERT’s vocabulary-agnostic semantic grounding and CharCNN’s character-level OOV handling. As a result, MeCoLog exhibits no instance of negative transfer across all evaluated pairs.

**Fig 8:**
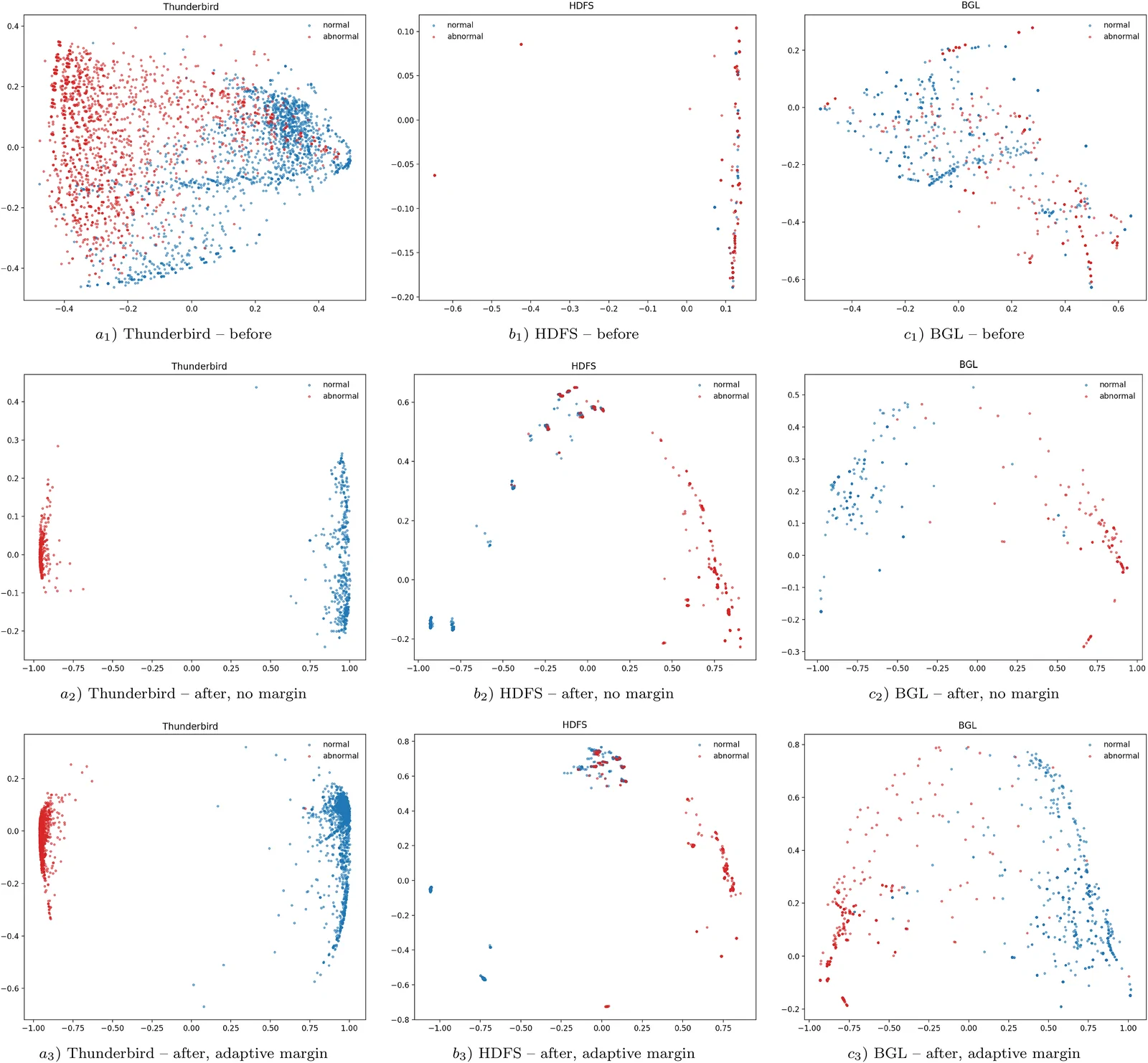
Log sequence representation before and after training phase for Thunderbird, HDFS and BGL dataset.

To further investigate the discriminative power of MeCoLog, we visualize the log sequence representations in the source systems across different training stages and ablation variants using t-SNE. As illustrated in Fig 8, there is a stark contrast between the initial feature distribution and the optimized embedding spaces. Initially ($a_{1},b_{1},c_{1}$), normal and anomalous sequences are heavily overlapped, making boundary detection challenging. When trained under the MeCoLog variant without margin ($a_{2},b_{2},c_{2}$), the model begins to separate the spaces, yet several hard anomalies remain loosely bound to the normal domain. Under the full APCL formulation ($a_{3},b_{3},c_{3}$), these hard anomalies are systematically displaced. This produces a well-separated normal cluster and a cleaner decision boundary. At the same time, the overall dispersion of anomalies is preserved compared to the no-margin version. This optimal separation is particularly pronounced in the Thunderbird dataset (*a*_3_), where normal logs are compressed into a dense cluster while anomalies are pushed to the periphery. Furthermore, the visualization reveals the intrinsic diversity of abnormal behaviors; instead of forming a single cluster, the anomalies are distributed across multiple sub-regions. This distribution reflects the model’s ability to capture various attack categories and arbitrary system failures without collapsing them into a single representation. It validates the effectiveness of the gated injection and the contrastive loss in preserving semantic richness.

To quantify this preserved dispersion beyond visual inspection, we measure intra-class dispersion of held-out embeddings using the mean pairwise cosine distance within each class. Under the full APCL, the dispersion among anomalies is 3.3–6.4 times larger than under a symmetric SupCon objective across the three source systems, while inter-class separation is preserved. This confirms that the multi-region structure observed in Fig 8 is not a visualization artifact: APCL avoids homogeneous anomaly compactness while still enabling prototype-based detection.

Table 4 shows the results of different methods when evaluated on the AIT-LDS v2.0 dataset. Overall, MeCoLog demonstrates exceptional performance on the AIT-LDS v2.0 dataset, establishing competitive state-of-the-art performance in cross-enterprise scenarios. Baseline methods often struggle with the diverse attack vectors and varying log distributions between different server environments. In contrast, MeCoLog maintains remarkable stability, with F1-scores and AUC values consistently exceeding 0.98. In the Russell Mitchell → Santos transfer task, MeCoLog achieves an F1-score of 0.989 and an AUC of 0.996, significantly outperforming competitive baselines such as MetaLog (0.946 F1) and LogRobust (0.901 F1). Similarly, in the reverse scenario (Santos → Russell Mitchell), the model reaches near-perfect Precision (0.997) and Recall (0.991). The performance gap is most evident when compared to traditional deep learning methods like DeepLog and LogAnomaly, which only manage AUC scores between 0.65 and 0.75. These results indicate that APCL effectively handles the diverse attack composition of the AIT dataset. It successfully distinguishes rare, sophisticated threats, such as webshell uploads and escalations, from high-volume normal server traffic.

**Table 4:** Performance comparison on the AIT-LDS v2.0 dataset across two cross-enterprise transfer scenarios, under the binary anomaly detection formulation.

Scenario	Metric	DeepLog	LogAnomaly	LogRobust	LogTAD	MetaLog	LogTransfer	LogFormer	MeCoLog
Russell Mitchell → Santos	AUC	0.721	0.652	0.931	0.794	0.969	0.925	0.971	**0.996**
	Precision	0.901	0.855	0.846	0.820	0.901	0.857	0.923	**0.996**
	Recall	0.850	0.971	0.964	0.869	**0.996**	0.911	0.978	0.983
	F1	0.875	0.909	0.901	0.844	0.946	0.883	0.950	**0.989**
Santos → Russell Mitchell	AUC	0.736	0.754	0.901	0.812	0.946	0.879	0.955	**0.981**
	Precision	0.948	0.939	0.928	0.791	**0.997**	0.817	0.889	**0.997**
	Recall	0.868	0.982	0.989	0.860	0.946	0.904	0.967	**0.991**
	F1	0.907	0.960	0.957	0.824	0.971	0.858	0.926	**0.994**

**Fig 9:**
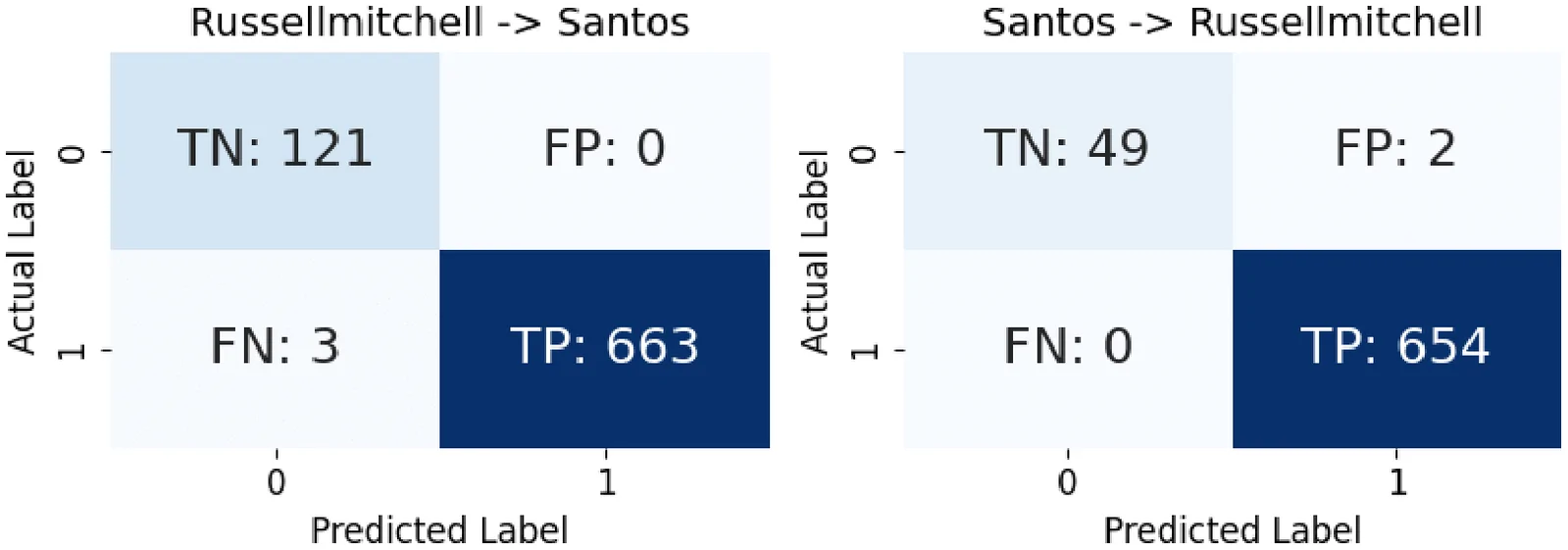
Binary confusion matrices on AIT-LDS v2.0, from random single run.

The near-perfect performance on AIT-LDS v2.0 stems from two factors. First, our cross-system evaluation trains and tests on distinct enterprises with completely isolated data partitions, eliminating any risk of data leakage from the sliding-window construction. Second, the task is framed as binary anomaly detection, where the model easily separates normal traffic from highly distinctive scanner signatures. Consequently, the high metrics reflect successful normal-versus-anomaly isolation rather than fine-grained multi-class attack attribution (e.g., identifying Escalate or Webshell Cmd), which remains a direction for future work. Fig 9 confirms this: only 3 false negatives out of 666 anomalies and 2 false positives out of 51 normal sequences are observed across both transfer directions.

### Few-shot sensitivity

**Fig 10:**
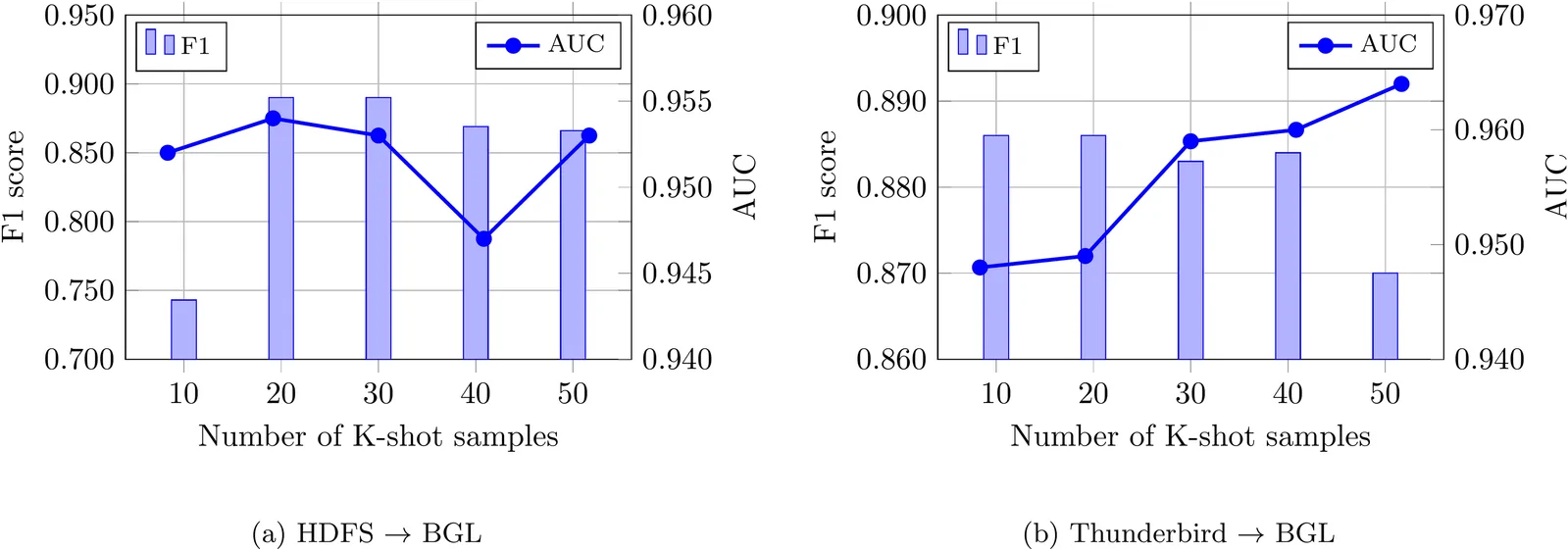
Sensitivity analysis of the number of *K*-shot samples. Bars indicate F1-score (left axis) while the line indicates AUC (right axis).

Fig 10 illustrates the sensitivity of MeCoLog to the number of K-shot support samples across two representative cross-system scenarios. Overall, the model exhibits remarkable stability even with an extremely limited supervision budget. In the HDFS → BGL task, we observe a significant performance leap when increasing *K* from 10 to 20, where the F1-score stabilizes around 0.89, suggesting that 20 samples provide sufficient structural context for the target system. Similarly, for the Thunderbird → BGL scenario, the AUC shows a steady upward trend, reaching its peak at *K* = 50. This sustained high performance across different *K* values confirms that MeCoLog’s asymmetric contrastive learning effectively leverages few-shot samples to align source-target representations without requiring exhaustive labeling.

### Ablation studies

Table 5 illustrates the performance impact of each proposed component within the MeCoLog method across two cross-system transfer scenarios. The Baseline in this table refers to MeCoLog without Parameter Integration, RoPE, or APCL, where the contrastive objective is replaced by a standard Prototypical Network (PN) loss. Overall, the results indicate a consistent performance degradation whenever a core module is removed. This confirms that each component plays a synergistic role in enhancing the model’s robustness and transferability.

**Table 5:** Ablation study: Contribution of each proposed component for MeCoLog.

Scenario	Method	AUC	Precision	Recall	F1
**HDFS** → **BGL**	Baseline (PN, no Param, no RoPE)	0.914	0.634	0.933	0.755
	MeCoLog fusion-additive	0.903	0.841	0.935	0.886
	MeCoLog fusion-concat	0.921	0.842	0.906	0.873
	MeCoLog w/o GKI	0.920	0.839	0.934	0.884
	MeCoLog w/o RoPE	0.921	0.659	0.933	0.772
	MeCoLog w/ SCL	0.933	0.632	0.930	0.753
	MeCoLog w/ APCL (no margin)	0.935	0.637	0.932	0.757
	MeCoLog w/ APCL (fixed margin)	0.935	0.701	0.932	0.800
	**MeCoLog (Full)**	**0.954**	**0.850**	**0.934**	**0.890**
**Thunderbird** → **BGL**	Baseline (PN, no Param, no RoPE)	0.857	0.834	0.725	0.776
	MeCoLog fusion-additive	0.919	0.859	0.867	0.863
	MeCoLog fusion-concat	0.926	0.857	0.885	0.871
	MeCoLog w/o GKI	0.899	0.856	0.837	0.847
	MeCoLog w/o RoPE	0.915	**0.860**	0.856	0.858
	MeCoLog w/ SCL	0.908	0.858	0.836	0.847
	MeCoLog w/ APCL (no margin)	0.912	0.860	0.859	0.860
	MeCoLog w/ APCL (fixed margin)	0.883	0.859	0.856	0.858
	**MeCoLog (Full)**	**0.949**	0.841	**0.937**	**0.886**

To further investigate the contribution of the GKI mechanism, we implement two alternative fusion strategies that replace the gated formulation in Eq. (4) while keeping all other components identical.

- *Additive fusion:* adds the parameter projection directly to the base key without a gate:
  $$\begin{array}{c} {𝐊}_{content}^{h}={𝐇}_{T}{𝐖}_{K}^{h}+LN({𝐇}_{P}{𝐖}_{P}^{h}). \end{array}$$ (15)
- *Concat fusion:* concatenates the base key and parameter projection, then applies a learned compression map ${𝐖}_{c}\in {\mathbb{R}}^{d_{model}\times 2d_{model}}$ to restore key dimensionality:
  $$\begin{array}{c} {𝐊}_{content}^{h}={𝐖}_{c}\;[{𝐇}_{T}{𝐖}_{K}^{h};\;LN({𝐇}_{P}{𝐖}_{P}^{h})]. \end{array}$$ (16)

Unlike GKI, neither variant can input-dependently attenuate the parameter contribution based on its content.

The removal of RoPE resulted in the most significant drop in F1-score for the HDFS → BGL task (falling from 0.890 to 0.772). This trend suggests that capturing relative temporal dependencies is important for distinguishing anomalous patterns in long-sequence distributed logs. Furthermore, the exclusion of Parameter Integration (w/o Parameter Integration) led to a noticeable decline in Precision, highlighting the importance of dynamic parameters in disambiguating log messages that represent different operational states.

To further disentangle the contribution of APCL, we evaluate two degraded variants of the loss function. *MeCoLog w/ APCL (no margin)* retains the APCL objective but removes the margin term for anomaly samples. *MeCoLog w/ APCL (fixed margin)* preserves the asymmetric contrastive structure but substitutes the adaptive margin with a fixed scalar. This removes the model’s ability to adjust decision boundaries based on inter-class distance. The performance gap between these variants and the full model confirms that both the contrastive asymmetry and the adaptive margin mechanism are necessary. Finally, replacing our contrastive strategy with SCL led to a decrease in AUC across both scenarios. This validates that MeCoLog’s asymmetric optimization maintains high discriminative power.

The fusion ablation results confirm that GKI outperforms both alternatives across both transfer scenarios. On HDFS→BGL, additive fusion achieves an F1 of 0.886 and concat fusion achieves 0.873, both below the full model (0.890). On Thunderbird→BGL, the same ordering holds (0.863 and 0.871 versus 0.886). The performance difference between additive fusion and GKI is modest in absolute terms but consistent across scenarios. This suggests that the content-dependent gate is the primary source of GKI’s contribution rather than the mere presence of a parameter pathway. Additive fusion always injects the full parameter signal regardless of its quality, whereas GKI can selectively suppress noisy embeddings such as those derived from IP addresses or hexadecimal identifiers on a per-event basis.

### Hyperparameter sensitivity

**Fig 11:**
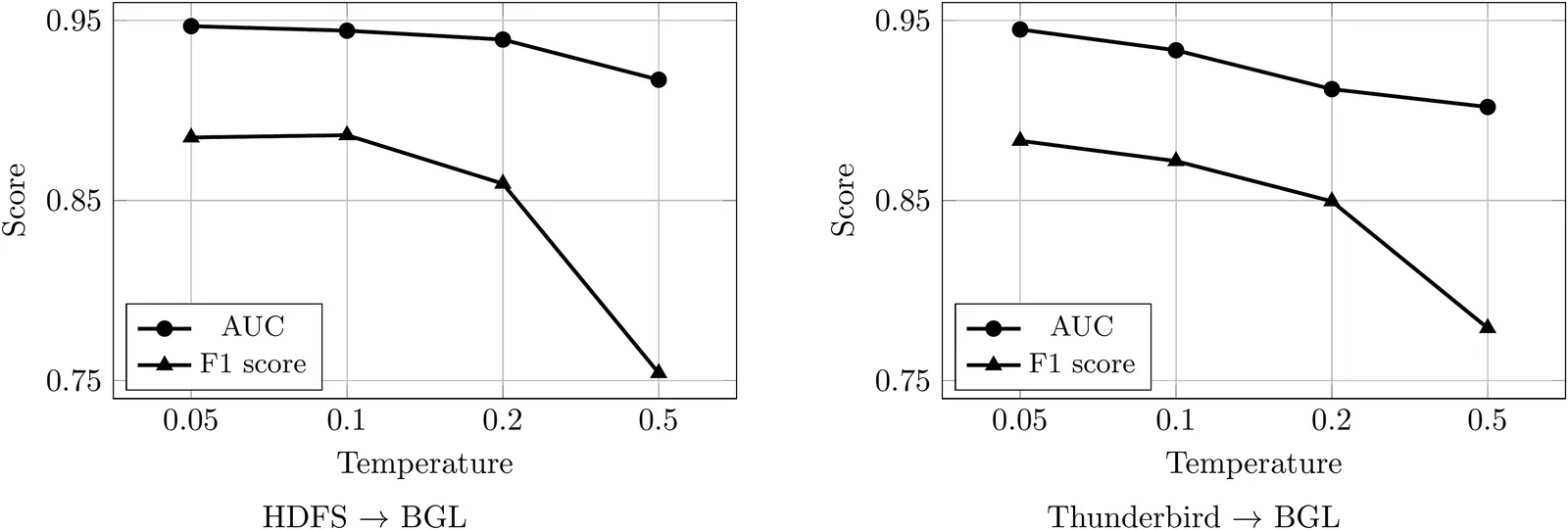
Effect of the temperature parameter on AUC and F1 score under two cross-system scenarios.

In this section, we evaluate the impact of the temperature hyperparameter τ on the model’s discriminative performance. The temperature parameter plays a critical role in contrastive learning by controlling the “hardness” of the distribution over negative samples. We conduct a grid sweep with τ∈{0.05,0.1,0.2,0.5} across two representative cross-system transfer pairs: HDFS → BGL and Thunderbird → BGL. The results are illustrated in Fig 11.

As shown in the figure, both the F1-score and AUC exhibit a high sensitivity to the choice of τ. We observe that lower temperature values (τ≤0.1) yield the best results, with performance peaking at approximately τ=0.05. Specifically, in the HDFS → BGL scenario, the F1-score reaches its maximum of 0.886 at τ=0.1, while the AUC remains robust at 0.946. However, as τ increases beyond 0.2, there is a sharp decline in detection accuracy, particularly in the F1-score which drops significantly to approximately 0.75–0.77 at τ=0.5.

This trend can be explained by the fact that a smaller temperature encourages the model to focus more on the most difficult negative samples (hard negatives), leading to a more compact and well-separated representation space. Conversely, a larger τ tends to smooth the similarity distribution, causing the model to treat all samples equally and losing its ability to distinguish subtle anomalies from normal operational patterns. These findings suggest that a sharper contrastive distribution is essential for maintaining high precision in cross-system anomaly detection tasks.

### Efficiency analysis

The theoretical complexity of MeCoLog remains competitive with standard Transformer architectures while incorporating rich, multi-modal log context. The Hybrid Parameter Integration layer operates with a complexity of $O(L\cdot M\cdot d_{c})$, where *L* is the sequence length, *M* is the number of parameters per log, and $d_{c}$ represents the character-level convolutional filter operations. Since *M* is typically a small constant, this stage scales linearly with the input size. The core Gated Transformer Encoder maintains the $O(L^{2}\cdot d)$ complexity of self-attention. The Gated Key Injection mechanism uses element-wise operations and linear projections, which do not alter the model’s asymptotic growth. Finally, the Prototypical Inference phase is highly efficient. It requires only a single forward pass to generate embeddings and an O(K·d) operation to compute the normal prototype from a support set of size *K*. Consequently, MeCoLog achieves superior detection capabilities without introducing the quadratic bottlenecks often associated with heavy cross-attention or fine-tuning procedures.

As shown in Table 6, all methods are measured under identical hardware and batch size (batch size = 1, sequence length = 20) on the same machine. For MeCoLog, the reported Inference Latency corresponds to the forward pass of the parameter-integrated Transformer encoder, including the CharCNN parameter branch. Parameter values are instance-specific and must be encoded at inference time. It excludes SBERT template encoding. SBERT is precomputed and cached as a frozen, shared feature extractor, and its output depends only on the log template rather than the specific instance.

**Table 6:** Efficiency comparison of MeCoLog and baselines on the AIT-LDS cross-system scenarios. Each range reports Russellmitchell→Santos and Santos→Russellmitchell. GPU memory is the peak CUDA reserved memory unless otherwise stated. GPU throughput is the batched model-only throughput measured under identical hardware.

Model	Training Time (s)	Inference Latency (s)	GPU Throughput (win/s)	GPU Memory (GB)	Parameters
MeCoLog	0.980–1.010	0.860–1.210	1766	0.865	8.34M
MeCoLog w/o GKI	0.730–0.820	0.760–0.850	2033–2037	0.865	7.09M
MeCoLog w/o RoPE	0.830–0.950	0.860–0.930	1765–1768	0.865	8.34M
DeepLog	6.550–6.670	0.730–0.830	949–965	0.328	0.23M
LogAnomaly	6.380–6.460	0.740–0.840	942–947	0.326	0.41M
LogRobust	7.000–7.130	**0.056–0.061**	12632–12957	0.959	0.56M
MetaLog	0.890–0.980	0.810–0.890	862–904	0.200	0.42M
LogTransfer	0.830–0.910	0.820–0.870	879–1008	0.100–0.200	0.29M
LogFormer	23.360–39.210	0.950–1.040	2321–2327	**0.137**	7.32M
LogTAD	7.480–12.780	**0.042–0.048**	**14688–18738**	0.525	0.36M

MeCoLog demonstrates a favorable balance between effectiveness and computational cost. Its training time is substantially lower than most conventional and transformer-based baselines and remains comparable to other meta-learning approaches. Although MeCoLog does not achieve the lowest inference latency, its runtime remains practical given its superior detection performance. In terms of GPU memory, MeCoLog requires a moderate amount of resources. Its ablation variants exhibit identical memory consumption, which indicates that Parameter Integration and RoPE introduce negligible overhead. The higher inference latency relative to lightweight baselines such as LogRobust (0.056–0.061s) is directly attributable to model capacity. MeCoLog’s 8.34M parameters, driven by the GKI mechanism, the CharCNN parameter encoder, and multi-head attention, provide richer representational capacity at the cost of higher per-sample latency. This remains practical for log monitoring deployments, which are not typically sub-millisecond latency-critical. Overall, despite having the largest parameter count among the evaluated methods, MeCoLog maintains efficient training and inference characteristics while providing strong cross-system generalization capability.

The throughput column contextualizes the per-sample latency. Under batched operation, MeCoLog sustains approximately 1,800 windows per second, within the same order of magnitude as LogFormer (approximately 2,300 win/s). LogFormer’s slightly higher throughput reflects the absence of the parameter encoder and GKI path. This corresponds to over 150 million windows per day on a single GPU. The batch-1 latency in the Inference Latency column therefore does not constrain realistic monitoring deployments.

## Conclusion

In this paper, we presented MeCoLog, a meta-contrastive learning method for few-shot cross-system log anomaly detection. By integrating a Hybrid Parameter Embedding layer with a Gated Key Injection mechanism, MeCoLog effectively captures the rich contextual semantics of log messages. Furthermore, the Asymmetric Prototypical Contrastive Learning (APCL) objective enables the model to align representations across disparate domains using only a handful of target samples, significantly reducing the manual labeling burden. Extensive experiments across various real-world datasets (including HDFS, BGL, Thunderbird, and AIT-LDS v2.0) demonstrate that MeCoLog consistently outperforms state-of-the-art baselines in cross-system transfer tasks, maintaining high robustness and efficiency under extreme few-shot constraints.

Despite these strengths, MeCoLog requires at least a few labeled target anomalies during adaptation and assumes source training systems have sufficient anomaly coverage. Additionally, our evaluation relies on standard benchmarks that may not fully capture the complexity of modern infrastructures. For future work, we plan to extend MeCoLog into a multi-source meta-training framework and develop a zero-anomaly adaptation variant via one-class classification fallback. We also aim to support online stream learning for real-time log shifts, evaluate the model on modern cloud and microservice environments, and explore the integration of Large Language Models (LLMs) to enhance the semantic understanding of rare failure modes.
